# Is It Quackery?

**Published:** 1878-08-20

**Authors:** 


### IS IT QUACKERY?

The following card has been sent us by a medical friend as emanating from a professed regular practitioner in Arkansas:

"DR. ———,

PRACTICING PHYSICIAN.

"*Special attention paid to Scrofula, Rheumatism, and Diseases of Women and Children. Syphilis permanently cured without mercury.*"

Our opinion is asked as to whether the card is ethical. The physicians in the place refuse to consult with the party, not only because the card itself is doubtful, but his conduct is otherwise unprofessional; and yet, inquiries touching his standing in other communities have been answered in his favor.

We hesitate not to say that the above card carries on its face unmistakable evidences of deliberate quackery. Those at a distance, who have recommended him, are, however, not necessarily involved in censure, as his past career may have conformed to the highest professional standards, while his removal to another field may have been for the express purpose of entering upon a career of quackery.

If, however, from personal friendship and *ring fellowship*, or like motive, an unprofessional act be indorsed or ignored by a member of the profession, or by a medical society, such member and such society is *particeps criminis*, and equally culpable with the offending party.

It is not surprising, when we consider the ignorance of the public—even of intelligent men—in regard to medical science, and the propensity of the people to accept the pretensions and pay their money to boastful and brazen-faced empirics, that men, weak in principle, should be found who yield to the temptation to enter the easier, and ofttimes, we are sorry to say, the more successful arena of fraud and deception.

As to whether specialties are or are not strictly ethical is a question upon which much difference of opinion exists in the profession. The code forbids them. But many good men in the profession advocate them as the best means of development and progress in the various branches of medical science. It being claimed that human life is too short, and the medical sciences too wide and extensive for any one man to do justice to the whole. When this question was sprung at the last meeting of the Georgia Medical Association, a majority seemed to hold the position that it is legitimate and proper for one who has first gone through with the entire curriculum of our colleges, and devoted himself for a time to the general practice, to take a specialty, and thoroughly perfecting himself therein, give exclusive attention to its study and practice. Every one cannot, of course, be specialists, but the general practitioner, it was claimed, would gladly have such specialist, to whom he could turn over cases which he was not himself able or prepared to manage, provided the specialist would treat him with due consideration and courtesy as the family physician. Such a specialist, confining himself to his legitimate branch, and having at hand all proper instruments and appliances for its proper and successful practice, and who does not travel from point to point, or advertise himself in unprofessional ways, is generally not only tolerated, but honored by the profession at large. We think it, therefore, probable that a specialism of the form just described will, at no distant day, be recognized and inculcated in the code of ethics.

